# From micropterism to hyperpterism: recognition strategy and standardized homology-driven terminology of the forewing venation patterns in planthoppers (Hemiptera: Fulgoromorpha)

**DOI:** 10.1007/s00435-014-0243-6

**Published:** 2014-09-21

**Authors:** Thierry Bourgoin, Rong-Rong Wang, Manfred Asche, Hannelore Hoch, Adeline Soulier-Perkins, Adam Stroiński, Sheryl Yap, Jacek Szwedo

**Affiliations:** 1 Département Systématique et Evolution, UMR 7205-ISyEB, MNHN-CNRS-UPMC-EPHE, Muséum National d’Histoire Naturelle, CP 50, 45 rue Buffon, 75005 Paris, France; 2 Institute of Zoology, Chinese Academy of Sciences, 1 Beichen West Road, Chaoyang District, Beijing, 100101 China; 3 Museum für Naturkunde Leibniz-Institut für Evolutions und Biodiversitätsforschung an der Humboldt Universität zu Berlin, AG Biosystematik Invalidenstr. 43, 10115 Berlin, Germany; 4 Department of Palaeozoology, Museum and Institute of Zoology, Polish Academy of Sciences, 64, Wilcza Street, 00-679 Warsaw, Poland; 5 Crop Protection Cluster and Museum of Natural History, University of the Philippines Los Baños, 4030 Los Baños, Laguna Philippines; 6 Department of Invertebrate Zoology and Parasitology, University of Gdansk, 59, Wita Stwosza Street, 80-308 Gdańsk, Poland

**Keywords:** Tegmina morphological patterns, Wing, Veins, Venation interpretation, Standardized terminology, Brachypterism, Fossil

## Abstract

Following recent advances in the morphological interpretations of the tegmen basal cell margins in the Paraneoptera, a standardized and homology-driven groundplan terminology for tegmina types, structures and vein patterns in Hemiptera Fulgoromorpha, including fossils, is proposed. Each term is listed with a morphological definition, compared and linked to the main systems of planthopper forewing description that have been reviewed. The importance of a standardized and homology-driven terminology is stressed to enhance the quality of data in taxonomic descriptions and to strengthen phylogenetic morphological analysis results. When the interpretation of the origin of vein branches is render difficult, a three-step strategy for pattern recognition of the vein is proposed based on two principles: (1) vein forks are more informative than topology of the vein branches: a search for homologous areas, the nodal cells in particular, must first guide the recognition rather the number of branches of a vein, and (2) minimum of ad hoc evolutionary events should be invoked in the understanding of a modified vein pattern. Examples of some conflicting interpretations of venation patterns in planthoppers are discussed within different families for both extant and extinct taxa. For the first time, the concept of brachypterism is defined in a non-relative way independently from other structures, and the new one of hyperpterism is proposed; a reporting system is proposed for each of them.

## Introduction

Hemiptera Fulgoromorpha, or planthoppers, constitute a large group of more than 13,000 obligatory phytophagous insect species distributed all around the world (Bourgoin 2014). Their etho-ecology is dominated by interactions with their host plants, which are not only sources of food but also oviposition and mating sites, shelter and a means to communicate (Nault and Rodriguez 1985; Denno and Perfect 1994). In 1987, over 150 species of planthoppers from various families were already directly or indirectly recorded as pests of 99 economic plants (Wilson and O’Brien 1987) and since, new invasive species as potential pests are regularly discovered. They include some of the most devastating pests of major agricultural crops throughout the world, several species vectoring a variety of plant pathogens such as phytoplasmas, viruses and other prokaryotes-like organisms (Wilson 2005). Reference systems for rigorous comparisons of data not only for correct species identification but also for more accurate phylogenetic analyses are therefore important to establish. Wing venation patterns and characters represent one of these conventional systems and are considered herein.

In arthropods, the ground plan of wing venation patterns consists in eight main veins divided in an anterior convex and a posterior concave branch, each dichotomously branched (Kukalová-Peck 1983, 1991). Between these main veins, secondary cross-veins occur. With evolution, this basic organization evolved with fusions, losses or additions of branches or veinlets, and with functional adaptations (Wootton 1992, 1996; Nel et al. 2012). However, in most cases, these changes have shaded the original organization and recognition of primary structures of the wing, making the evaluation of the homology of the venation and forewing structures between taxa a real challenge.

In planthoppers, venation characters have been established and extensively used for recognition as diagnostic characters over the last 100 years at all levels of classification: from single species descriptions to tribe or familial recognition and obviously particularly for description of fossil taxa (Metcalf 1913; Muir 1913, 1923; Comstock 1918; Melichar 1923; Fennah 1944; Hamilton 1972; Emeljanov 1977, 1987; Shcherbakov 1981, 1996; Zelazny 1981; Anufriev and Emeljanov 1988; Dworakowska 1988; Bourgoin 1997; Zelazny and Webb 2011). They have also been used in some recent morphological phylogenies, but only in few taxonomic units such as Delphacidae (Asche 1985), Kinnaridae-Meenoplidae (Bourgoin 1993), Cixiidae (Ceotto and Bourgoin 2008) and Lophopidae (Soulier-Perkins 2001; Soulier-Perkins et al. 2013).

While in general it is relatively easy to recognize and name the veins in most planthopper taxa, this becomes a difficult task in some of them due to factors such as:

1. Branch veins reductions or polymerizations, vein anastomosis, specializations, early or late vein forks, vein-like structure (e.g. ‘arculus’, Wootton 1992), false veins (Attié et al. 2002) and veinlets mimicking true veins (Szwedo and Żyła 2009), with some of these probably linked to modifications to strengthening the wing for flight (Wootton 1996; Nel et al. 2012);
2. Forewing modifications that have been variously labelled with submacropterism, subbrachypterism, brachypterism, eubrachypterism or micropterism, according to their development or also as coriaceous, coleopterous, koeliopterous (coeliopterous) forms (Szwedo et al. 2013) according to their apparent structure;
3. More general morphological adaptations to specialized habitats/behaviour, such as dipterization (Rohdendorf 1943) or the recently described issidization (Gnezdilov 2013);
4. Some specialized evolution of a few taxa such as stenopterism (Gnezdilov 2012a, b).

Each of these factors has sometimes significantly altered the general venation pattern that then becomes hardly identifiable. They have, moreover, generated different views on the interpretation of the veins (and therefore of their underlying homologies) that led to divergent terminologies used to describe them (Emeljanov 1994; Bourgoin 1997). However, a homology-based recognition of these characters translated into a standardized terminology is the basis of a coherent taxonomy of species recognition and description, is fundamental to morphological phylogenies and is crucial to relate the current and fossil taxa when only tegmina characters are available to explore and formulate evolutionary scenarios.

This absence of an obvious consensus for a standardized terminology of the forewing structures and veins has made it difficult to use them in further global evolutionary analysis. It also has incidentally contributed to the idea that wing structures are much too variable for morphological phylogenetic analyses. Subsequently, to be used, it asks for prior re-interpretation of the wing character homologies into a single knowledge system for not using different characters under the same name and vice versa.

The purpose of the paper is therefore (1) to review the various interpretations and vein nomenclature systems that have already been used in Hemiptera Fulgoromorpha, (2) to propose a standardized homology-driven terminology with its definitions, which will be shared and used in future taxonomic descriptions and morphological phylogenies that will use tegmen characters and (3) to propose a three-step strategy for vein patterns recognition when the interpretation of vein branches is too obscured, illustrated by current conflicting interpretations in some examples. As brachypterism or micropterism defines wing reduction, we also recognize hyperpterism (wing hyper-development) and we propose a reporting system to document them in a non-relative way.

## Material and method

As elytra is used in Coleoptera, we used the term ‘tegmina’ (singular: tegmen) as a synonym to mention the more or less sclerified mesothoracic forewings, a convention in most of Hemiptera; they are usually covering the membranous metathoracic hind wings at repose.

The general venation schema for planthoppers is here provided based on a fulgoromorphan ground plan slightly modified from the one proposed by Shcherbakov (1996). Terminology is completed according to Bourgoin (1997) who recommended the use of areas (nodal cells, major vein areas) for the interpretation of veins and updated from Bourgoin and Szwedo (2008) and Szwedo and Żyła (2009), including the recent proposal of the CuA zigzag vein (=arculus *auctorum*, Emeljanov 1987) as autapomorphic for Paraneoptera (Nel et al. 2012).

The standardized terminology proposed is built upon the various major vein nomenclature systems used and upon homology-driven morphological interpretations concerning both extant and extinct taxa samples according to all major authors in these topics (Metcalf 1913; Muir 1913, 1923; Melichar 1923; Fennah 1944; Hamilton 1972; Emeljanov 1977, 1987; Shcherbakov 1981, 1996; Zelazny 1981; Kukalová-Peck 1983; Chou et al. 1985; Anufriev and Emeljanov 1988; Dworakowska 1988; Bourgoin 1997; Zelazny and Webb 2011; Ding 2006; Nel et al. 2012, 2013; Gnezdilov 2013).

A corresponding terminology between these major systems is proposed (Table 1), and a definition is provided for each structure.

**Table 1:** Corresponding terminologies between main vein system interpretations since Metcalf (1913), with the recommended standardized one (in bold)

Metcalf (1913)	C		Sc + R + M	Sc + R	Sc (two branches)		R	M (typically 4 branched)	Cu	A1	A2	A3	A3 s branch
Melichar (1923)	Costal vein			Subcostal vein		Radius 1	Radius 2	Median vein	Cubitus	Clavus suture	Claval vein external branch	Claval vein internal branch	Scutellar + clavus sutural margins
Fennah (1944)	C		Sc + R + M	Sc + R	Sc		R1, Rs	MP	Cu1	Cu2	Pcu	A1	A2
Hamilton (1972)	C	Sc	S + M	S		SA	SP	M (forks first in MA and MP)	Cu	P + E	1A	2A	Margin
Emeljanov (1977)	C	Sc	S + M	Sc + R		R_1_Sc + R_2_	R_3_	M	CuA	CuP	Pcu	A1	A2
Shcherbakov (1981)	C		Sc + R + M	(Sc +) R	R_1_	R_1_a	Rs	M	CuA	CuP	Pcu	A1	A2
Chou et al. (1985)	C	Sc	R + M	R				M	Cu	Clavus suture	A	A	Margin
Dworakowska (1988)	CA	Pc + CP	ScP + R + M	ScP + R + MA	ScP + RA	RA	RP + MA	MP	CuA	CuP	AA	AP’	AP”
Ding (2006)	C		Sc + R + M	Sc + R	Sc	Sc2	R_1_	M (‘Rs, M_1_, M_2_, M_3_′ = M_1_, M_2_, M_3_, M_4_)	Cu1 (‘Cu1a’ = CuA_1_)	Cu2	IA	IIA	Margin
**Interpretation and recommended terminology**	**CA**	**Pc** **+** **CP**	**ScP** **+** **R** **+** **M** **+** **CuA**	**ScP** **+** **R**	**ScP** **+** **RA**	**RA**	**RP** (+MA)	**MP**	**CuA** (forks in CuA_1_ and CuA_2_)	**CuP**	**Pcu**	**A1**	**A2**

## Results

### Tegminal veins

**Fig. 1:**
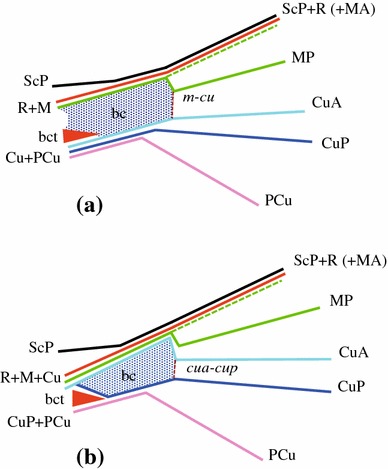
Schematic representation of the basal cell margins in a Fulgoromorpha tegmen according to the classical interpretation (**a**) and according to Nel et al. (2012) (**b**) with the paraneopteran autapomorphic CuA zigzag vein and the basal apomorphic fusion of R, M and CuA. *bc* Basal cell, *bct* basicubital triangle, veins nomenclature as in text

**Fig. 2:**
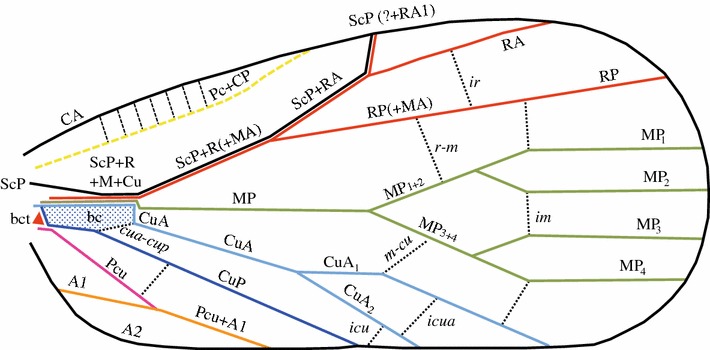
General venation schema of a Fulgoromorpha tegmen (adapted from the ground plan proposed by Shcherbakov (1996) for planthoppers)

Recently, Nel et al. (2012) proposed a new interpretation of the Paraneoptera wing base with the fusion in a common stem of the bases of three veins: the radius (R), the media (M) and the cubital anterior (CuA). This basal fusion plus the presence of a specialized basal cross-vein *cua*–*cup* are two apomorphies that purport to support the monophyly of the Paraneoptera. We follow here this interpretation with a *cua*–*cup* veinlet closing anteriorly the basal cell (Fig. 1b) versus an *mp*–*cu* veinlet (Fig. 1a) as in the classical interpretation. Vein tegmina terminology in planthoppers is summarized accordingly in Fig. 2.

*Costal* *margin* It represents a complex of veins, it could be formed by the single vein costa anterior (CA) or most often it is composed by the veins CA and the fused precosta + costa posterior (Pc + CP), as proposed by Dworakowska (1988) using the data and interpretations of Kukalová-Peck (1983).

*Precosta* + *costa* *posterior* (Pc + CP) It is a complex of two veins (Dworakowska 1988: Figs. 1–12) often fused completely, sometimes partly or to certain extent with CA or shifted from the costal margin for a distance along the costal margin (=C for Handlirsch (1922) and =Sc for Martynov (1926) in the fossil Fulgoridiidae genus *Fulgoridium* Handlirsch).

*Subcosta* *anterior* (ScA) ScA is considered as reduced in Paraneoptera (Kukalová-Peck 1991; Nel et al. 2012).

*Subcosta* *posterior* + *radius* (ScP + R) They represent another complex of veins fused shortly after their base. ScP is basally independent and joins distally the anterior margin of the basal cell formed by the common stem of R + M + CuA (Fig. 1b). ScP + R usually forked medially into the subcosta posterior + radius anterior branch (ScP + RA) and the radius posterior branch (RP), the latter sometimes still named *sector radii* (Rs) following the Comstock–Needham system (1899a, b, c). Sc + RA forks distally into ScP and RA_1_, and the following branches are numbered subsequently, RA_2_, RA_3_, etc. Sometimes, the branches Sc + RA and RP separate early, even directly at the basal cell level (e.g. in some Tropiduchidae genera such as *Thymbra* Melichar, 1914, *Montrouzierana* Melichar, 1912 or Alcestini Melichar).

*Media* (M) Among the Hemiptera, this vein is in fact only homologous to the *media posterior* (MP) as the vein *media anterior* (MA) is considered to remain fully fused with the RP branch (Fig. 1b) (Kukalová-Peck 1991; Nel et al. 2012). It separates from the common stem ScP + R + M + CuA generally at the distal margin of the basal cell. However, this point of separation is variable as MP individualizes sometimes from a short common stalk with Sc + R or even from a common stalk with CuA. The first forking of MP is its division into MP_1+2_ and MP_3+4_ branches. It is an important landmark that has generated confusion (Fig. 6); however, in a few cases, the branches MP_1+2_ and MP_3+4_ might leave the basal cell separately (e.g. some Ricaniidae species as in genera *Ricania* or *Pochazia*).

**Fig. 3:**
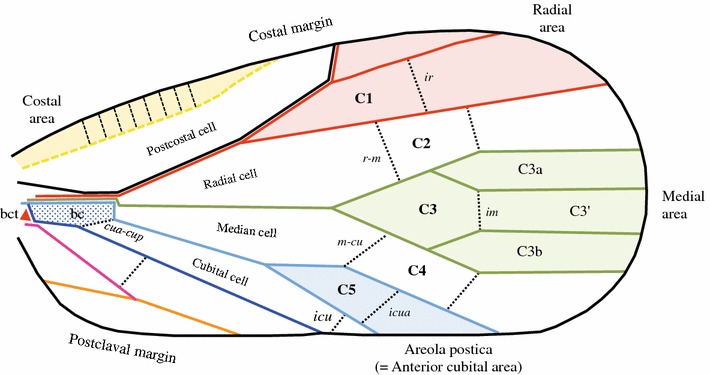
Schematic representation of a Fulgoromorpha tegmen: basal and nodal cells, vein areas

*Cubitus* *anterior* (CuA) It is the last branch leaving the common stem ScP + R + MP + CuA according to the model proposed by Nel et al. (2012) (Fig. 1b). It forks into CuA_1_ and CuA_2_ branches, delimitating the *areola postica* (Hennig 1981).

*Cubitus* *posterior* (CuP) It is a vein corresponding to the claval suture *auctorum,* claval vein or *vena dividens* [=A1 of Martynov (1926)]. It never forks and usually reaches the posterior margin of the tegmen delimiting anteriorly the clavus.

*Postcubitus* (Pcu) It is the first vein on the clavus (Emeljanov 1987; Anufriev and Emeljanov 1988). It is always apically fused with first anal vein in Fulgoromorpha (but not exclusively) to form a common apical stem Pcu + A_1_ reaching the apex of clavus or the claval margin or CuP, both known as the Y-vein.

*First* *anal* *vein* (A_1_) The second vein on clavus, part of the Y-vein, fused with Pcu to form a common stalk reaching the claval margin or the apex of clavus or CuP.

*Second* *anal* *vein* (A_2_) This vein forms the claval margin on the tegmen.

Between these longitudinal veins (always noted with capital letters), a network of transversal veinlets (cross-veins) links the main veins. They are conventionally noted in lowercase letters and generally in italics. This transversal network appears to be much more diverse than the veins. Often, veinlets more or less align to form a transverse line at the nodal level. Veinlets occurring basal to this ‘nodal line’ are usually good landmarks, and they appear less reliable distally, although they can form ‘postnodal lines’ or other structures of taxonomic/phylogenetical interest.

*cua* **–** *cup* *veinlet* This special veinlet, putatively definitive of Paraneoptera, closes the basal cell between stems CuA and CuP (Fig. 1b) according to Nel et al.’s (2012) interpretation.

*Nodal* *line* This is a virtual line composed of short segments of veins and veinlets more or less aligned and separating the corium from the membrane. It starts in the pterostigma area at the *nodus* (an imprecise term that should be abandoned) near where ScP or RA meets the tegmina margin and ends at the apex of the clavus. This line is a functional structure of bending (i.e. flexion, Wootton 1996), related to the mechanical properties of the tegmen, very often separating a stiffer corium from a more membranous membrane, which is well visible on SEM photographs (Fig. 5c). One or several postnodal lines or subapical lines have been also described in some taxa (see further: ‘hyperpterism’).

*Peripheral* *membrane* A special and very narrow marginal area, extending from the nodus to the apex of the clavus; when present, it forms a more or less radially undulated fringe delimitating marginally the membrane of the tegmen.

*Postclaval* *margin* (=*tornus*) It corresponds to the tegmen margin between the apex of the clavus and the proximal posteroapical angle of the tegmen (claval angle).

### Tegminal areas

**Fig. 4:**
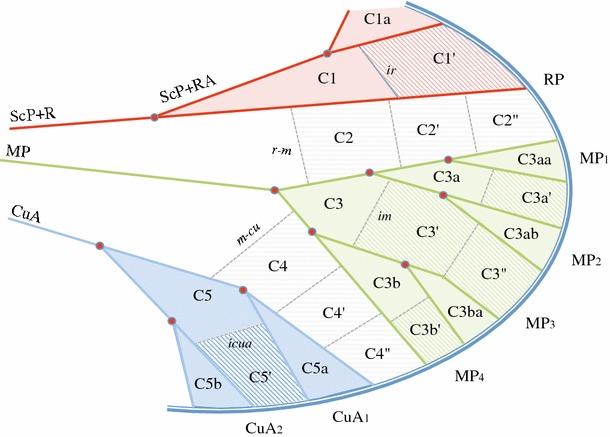
Nomenclature of nodal and postnodal cells for a Fulgoromorpha tegmen

Cells and areas (Figs. 3, 4) form complex characters that are useful for taxonomic description and, if carefully analysed (in term of homologies), that can also be used in phylogenetic reconstructions. There are five pre-nodal cells: one basal cell and four cells named according to their anteriorly bordering vein (postcostal, radial, median and cubital cell). The nodal (C1–5) and postnodal cells (terminology in ‘a’ and ‘b’) are named after the model proposed by Bourgoin (1997) and Bourgoin and Szwedo (2008). Areas are named after the model proposed by Szwedo and Żyła (2009). Cells are said to be ‘open’ when one of their sides corresponds with the margin of the tegmen, and they are said ‘closed’ when they are fully delimited by vein branches and veinlet(s) (Comstock and Needham 1899a, b, c).

#### Prenodal cells

*Basal* *cell* (bc) The area delimited by the basal portion of common stem R + M + CuA (plus ScP joining R distally) anteriorly, MP + CuA and *cua*–*cup* veinlet distally, and CuP postero-proximally (Fig. 1b). In this interpretation, a single origin is retained for the original trunk of the cubital vein, which is considered to fork very early into CuA and CuP branches. The basal cell is therefore a fully cubital area (between CuA, CuP and *cua*–*cup*) anterior to the basicubital triangle, which was not explicit in Nel et al. (2012). It is short and generally truncated in Fulgoromorpha.

*Postcostal* *cell* First basal cell between Pc + CP and anterior to ScP (or ScP + RA). Open cell or closed distally by a more or less transverse veinlet (=‘costal cell’ of Szwedo and Żyła 2009).

*Radial* *cell* Area between stems Sc + R and MP, proximally delimited by the basal cell and distally by the first *r*–*m* veinlet (=‘anterior discal cell’ of Szwedo and Żyła 2009).

*Median* *cell* Area between stems MP (and/or MP_3+4_ and/or MP_4_) and CuA (and/or CuA_1_), proximally delimited by the zigzag portion of stem CuA (Nel et al. 2012) (=arculus *auctorum*) and distally by the first *m*-*cua* veinlet (=‘posterior discal cell’ of Szwedo and Żyła 2009).

*Cubital* *cell* Cell anteriorly limited by CuA and posteriorly by CuP, proximally by the transverse *cua*–*cup* of the basal cell and distally by the tegmen margin (open cell) or by a cu-margin transverse veinlet.

#### Nodal cells

*Cell* *C1* (C1) Area delimited by the forking of ScP + RA and RP and distally closed with the inter-radial transverse veinlet *ir* between the branches ScP + RA (or RA) and RP (=‘outer anteapical cell’ of Szwedo and Żyła 2009).

*Cell* *C2* (C2) Area between the branches ScP + R and MP/MP_1+2_, proximally and distally delimited by the radio-medial transverse veinlets *r*–*m*_1_ and *r*–*m*_2_, respectively.

*Cell* *C3* (C3) Area between the first forking of stem MP, i.e. branches MP_1+2_/MP_2_ and MP_3+4_/MP_3_, distally closed by an inter-median transverse veinlet *im*. Cells C3a and C3b are the first cells, respectively, formed between MP_1_ and MP_2_, and MP_3_ and MP_4_. They are generally open cells but might be also often closed by veinlets. When present, a cell C3′ is distal to C3.

*Cell* *C4* (C4) Area between stem MP or its most posterior branches (MP_3+4_ or MP_4_) and stem CuA/CuA_1_, proximally and distally delimited by the mediocubital transverse veinlets *m*-*cua*_*1*_ and *m*-*cua*_*2*_, respectively.

*Cell* *C5* (C5) In the areola postica, the cell delimited by the first fork of stem CuA, i.e. branches CuA_1_ and CuA_2_, distally delimited by the intercubital transverse veinlet *icua* (=‘procubital cell’ Emeljanov 1994). This cell might become virtual or absent by anastomosis of the 2 CuA branches (=procubital cell ‘closed’ Emeljanov 1994).

#### Tegmen areas

*Costal* *area* Area delimited by the veins CA and Pc + CP. It could be absent (when CA and Pc + CP fused), narrow, wide, with or without transverse veinlets all along or in its distal part only, often more or less sclerotized. An area that probably evolved independently several times in planthoppers.

*Radial* *area* Area delimited by the anterior and posterior branches of stem Sc + R, up to margin. Proximally starting with C1.

*Medial* *area* Area delimited by the anterior and posterior branches of stem M, up to margin. Proximally starting with C3.

*Areola* *postica* (Hennig 1981) The area between CuA_1_ and CuA_2_ branches, up to the margin, proximally enclosing C5.

#### Nodal and postnodal cell nomenclature (Fig. 4)

Nodal cells differ as being born from a basal fork of a vein: C1 from the basal fork of Sc + R, C3 from M and C5 from CuA, or from a delimitated area normally intercalated between two veins and two veinlets: C2 between RP and M, *r*–*m*_1_ and *r*–*m*_2_, and C4 between M and CuA, *m*-*cua*_*1*_ and *m*-*cua*_*2*_. Similarly postnodal cells are also of two kinds: those born from a second fork of the vein and those which are intercalated cells between branch veins. The second fork give rise to postnodal cell named with (a) (=fork concerning the anterior branch of the previous fork) and (b) (fork concerning the posterior branch of the previous fork), such as for cells C3a, C3b, C4a, C5a and C5b. Intercalated cells are named with the prime symbol (′) (such as C3′, C5′). If necessary for a description, cells generated by the third fork will be noted ‘aa’, ‘ab’, ‘ba’, ‘bb’, etc., and the next intercalated cells with the double prime symbol (″). Figure 4 illustrates this nomenclature that use the fork (homologous landmarks) of the veins rather that the numbering of the veins (topology) as the first criteria of homology recognition. Due to an inversion of the drawing, Szwedo and Zyla (2009: Figs. 9, 10) mislabelled C5a for C5b. Indeed, C5a is absent in *Aulieezidium karatauense* Szwedo and Zyla (2009) (CuA_1_ remains unbranched) and present as an open cell in *Fulgoridium balticum* (Geinitz 1880) where CuA_1_ is 3 or 4 branched before reaching the tegmen margin.

### Other tegminal structures

*Basicubital* *triangle* (bct) A more or less triangular sclerotization at the base of the tegmen, between the posterior margin of the basal cell represented by CuP and the very basal portion of Pcu; it is sometimes hardly visible, very short in Fulgoromorpha (Shcherbakov 1996).

C*orium* Excluding the clavus and restricted to the proximal part of tegmen, relatively to the nodal line (*contra* Melichar 1923) and posteriorly delimited by the claval suture (vein CuP).

*Membrane* The distal part of tegmen relatively to the nodal line, i.e. postnodal portion of tegmen.

*Remigium* The corium plus the membrane area.

*Clavus* The part of the tegmen delimited by veins CuP (claval suture) and A_2_ (posterior margin of tegmen). The clavus is said to be ‘open’ when CuP does not reach the claval margin (A_2_) but merges with an anterior CuA branch as in most Achilidae, some Derbidae, or when CuP is weakened apically and not reaching margin as in extinct Mimarachnidae; it is said to be ‘closed’ when it reaches the claval margin distal to the Y-vein (Pcu + A_1_) joining A_2_. The open clavus can occur in different non-homologous ways in planthoppers.

*Pterostigma* An homoplasic and diversified sclerotized, and usually darkened, area of the tegmen that may include the apical portion of costal vein and/or ScP + RA vein and/or portion of the peripheral membrane.

*Versteifung* A sclerotized process on the ventral side of the tegmen, near or on the brace *cua*–*cup*; the Versteifung is a reinforcement corresponding to an attachment system to the thorax or an adjusting device for the hind wings in repose (Haupt 1929; Heslop 1955; Nel et al. 2012).

*Wing*-*coupling fore fold (WCFF)* In most (but not all) planthoppers, a longitudinal fold along the claval margin of the tegmen forming the mesothoracic part of the wing-coupling apparatus and connecting with a corresponding fold, lobe or hook in the costal margin of the metathoracic wing during the insect flight (d’Urso and Ippolito 1994).

### Tegmen size modifications

*Brachypterism, koeliopterism, macropterism, hyperpterism* Brachypterism is well known in planthoppers (Metcalf 1950), and it has been documented/discussed in various taxa such as Coleoscytidae (Bourgoin and Szwedo 2008), Delphacidae (Asche 1985), Ricaniidae (Stroiński et al. 2011) and Tropiduchidae (Asche and Wilson 1989; Huang and Bourgoin 1993; Gnezdilov 2012a, b). It can be more or less pronounced as referred by various terms that, in fact, do not apply to the tegmina alone but together to the hind wing development. Indeed, these terms define more a general state of the insect (macropterous, submacropterous, subbrachypterous, brachypterous or eubrachypterous forms) than they describe the structure itself (e.g. in Heteroptera Nabidae Kerzhner 1981). Previously, Metcalf (1950) defined macropterous tegmina as usually longer than the abdomen and koeliopterous tegmina as those of moderate length, covering most of the abdomen and with fairly developed venation.^1^

However, all these terms remain in fact subjective, sometimes mixed (e.g. brachypterism with micropterism), not enough indicative and descriptive of the tegmina when reductions or hyper-developments occur. They indeed cover a wide range of different situations that appeared particularly difficult to analyse objectively and precisely in comparative studies. We suggest therefore here new definitions for a new system of recognition of the degree of tegmina development in planthoppers that recognizes both brachypterism and hyperpterism concepts in a more objective way, the latter proposed as a new concept.

*Macropterism* Normal condition; supposed to be represented in the ground plan of the Fulgoromorpha (Fig. 1), with a transversal row of 5–6 closed nodal cells, plus one complete distal row of open and closed postnodal cells (generally at least C3′) (Fig. 5a). Many variations should happen around this ground plan with anastomosing or polymerization of veins, earlier forks of veins at the basal cell level or even no fork, e.g. simple CuA in the issid genus *Oronoqua* Fennah, 1947 (Gnezdilov et al. 2010).

*Brachypterism* Expressing various degrees of non-proportional shortening of the tegmen with impoverishment of the venation leading to observe open nodal cells (most communally C5, C4 and/or C2) and with veins tending to remain unforked. Brachyptery might occur independently in various areas of the wing, leading to different tegmina general shapes. When describing brachypterous species, we suggest adding the open nodal cell(s) for a clearer descriptive terminology of the tegmina as follows: brachypterous in Cn—with Cn referring to the open nodal cell(s) concerned. As an example in Fig. 5b, *Coleoscyta rotundata* Martynov (Fulgoromorpha, Coleoscytidae) is defined as brachypterous in C1–5.

Accordingly and comparatively to other taxa, many delphacid taxa should be considered as brachypterous with cells C2, C3 and C4 open. In some species, brachyptery is so pronounced that even the nodal line has disappeared and nodal cells are just missing. We suggest to specify therefore the open prenodal cells: the delphacid *Conomelus lorifer dehneli* Nast (Asche 1985, Fig. 250c) is for instance described as brachypterous in postcostal, radial, median and cubital cells.

*Hyperpterism* This new concept expresses various degrees of hyper-development of the tegmina from the fulgoromorphan ground plan with addition of supranumerous forkings of main veins (more than 2) leading to recognize at least a second rank of postnodal closed cell(s) after the nodal cells. The concept only considers degree of branching and is not related to wing size. As brachypterism, hyperpterism may occur independently in different areas of the tegmen and in various families, such as in a non-described Tropiduchidae genus (Fig. 5c); it might also be characteristic for higher taxa, such as in Fulgoridae, Dictyopharidae, Derbidae, Ricaniidae and Lophopidae (Fig. 5d) for instance. When describing hyperpterous species, we suggest adding the veins names concerned by the supranumerous forks. In Fig. 5c, the tropiduchid is hyperpterous in R and M but not in CuA (only two forks for each branch); in Fig. 8d, the derbid *Zoraida (Neozoraida) ugandensis* Distant, 1914, is hyperpterous in MP; and in Fig. 5d, the Lophopid *Magia* is hyperpterous in R, MP_1+2_ and CuA.

*Micropterism* Often mistaken for brachypterism, it documents a phenomenon of dwarfing or miniaturization (proportional shortening) of the tegmina, with a near complete venation pattern, and in one taxon comparatively to related taxa in the same taxonomic group.

*Stenopterism* Straightening of the basal part of the tegmina, often with the basal fusion of the mains stems of the longitudinal veins and reduction of clavus; often linked to dipterization (Waterhouse 1839; Rohdendorf 1943; Fennah 1949; Gnezdilov 2012a, b) as in Derbidae or some Tropiduchidae Gaetuliini.

Besides these terms, several other terms have been used in tegmen description such as membranous, translucid versus opaque, or coriaceous, coleopterous, coeliopterous. While they may be useful in the diagnosis of a species, they are useless to describe them precisely and with little morphological value.

## Discussion

An absence of standardization in vein terminology may affect the correct use of identification keys and the identification of taxa. It may also restrict the correct recognition of homology between vein characters and then the phylogenetic reconstruction of taxa, the communication and good understanding between scientists and more generally efficiency of scientific result diffusion.

Generally, conflicting terminologies occur in species or genera with specialized tegmina that makes it difficult to recognize the usual landmarks. While they should be solved correctly, these conflicts have limited effect on the general understanding of the evolution of the group. Sometimes, however, these occur at a higher level of the classification, in various families, and might directly affect phylogenetical reconstructions of the taxa that have been interpreted under different systems.

In Delphacidae for instance, Ding (2006: Fig. 6) proposed a venation scheme for the family where M firstly forks into a long M_1_ largely fused with Rs and the other branches of M. M_3_ is also largely fused with CuA_1_, and M_4_ is absent. Accepting such a system and interpretations and therefore the underlying homologies would bring several new autapomorphies for the family and would invoke a series of new and ad hoc evolutionary events to explain their evolution within the planthopper framework. However, a simpler scheme with a single Sc, a single forked R at the nodal line (sometimes shortly fused with M_1+2_ as in the genus *Sogatella* Fennah for instance), and a long unforked M_3+4_, would perfectly fit with the classical ground plan of the planthoppers. Similarly, different terminologies due to conflicting vein interpretations were also reported in the family Meenoplidae (Bourgoin 1997) with identical consequences regarding the homology of tegmina characters, the family for phylogenetical reconstruction (Bourgoin 1993).

However, sometimes the venation pattern is so altered that its recognition remains problematic resulting in several interpretative hypotheses. In these cases, and when being without other evidence, Bourgoin (1997) has proposed a strategy minimizing ad hoc hypothesis to interpret homologies of a modified venation pattern by using a parsimonious approach based on a hierarchy of the evolutionary events advocated. It follows two principles: (1) vein forks are more informative than topology of the vein branches: search for homologous landmarks and areas, such as the nodal cells in particular, should first guide the recognition rather the number of branches of a vein, and (2) minimum of ad hoc evolutionary events should be invoked in the understanding of a modified vein pattern. We complete here these views, and we propose a hierarchical three-step process of recognition:

1. Vein forks are more important homological landmarks than vein branches topology and their number, and they should be looked for first to deduce the vein recognition. Figure 6 illustrates this step for the median forks. The general pattern (Fig. 6a), as observed in most planthoppers, is a first fork (A) that separates the branches M_1+2_ and M_3+4_, each separating again into two branches. In some taxa, a superficially similar pattern is often observed with four branches of M but generated by a different forking system (Fig. 6b). Focussing first on branch topology and number would lead to recognize a common stem M_1+2+3_ absent in the general planthopper tegmen pattern plus a nodal cell C3 bordered by M_2_, M_3_ and M_1+2_ (Fig. 6c) but missing its M_4_ margin, thus an area non-homologous with the other planthopper C3. Interpretation as in Fig. 6d would be therefore preferred.

**Fig. 5:**
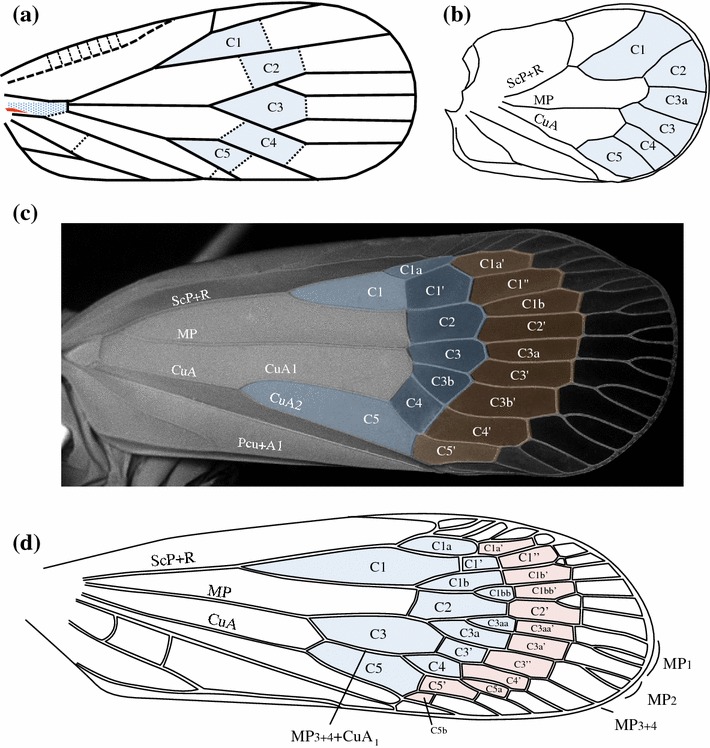
Tegmina development: **a** macropterous form, schematic representation with a full raw (*blue shaded*) of closed nodal cells. **b** Brachypterous form as in *Coleoscyta rotundata* Martynov, 1935 (Fulgoromorpha, Coleoscytinidae), with absence of closed nodal cell, (redrawn from photo in Szwedo et al. 2004). **c** Hyperpterous form of a non-described new genus of Tropiduchidae, with an additional raw of closed postnodal cells (*red shaded*). **d** Hyperpterous form of *Magia sp*. (Lophopidae) with an additional raw of closed postnodal cells (*red shaded*)
2. Continuity of veins to the wing margin should be retained versus its distal interruption before the margin and branch vein fusing with another branch should be tested as a first interpretation before branch vein vanishing. For instance, RA and MP have always been interpreted as remaining fully fused in Hemiptera (Kukalová-Peck 1991; Nel et al. 2012), but never the loss of RP. It is also valid for any other apical branches where a simple merging with an adjacent vein is preferred to the distal vanishing of the vein (Fig. 7b–d).

**Fig. 6:**
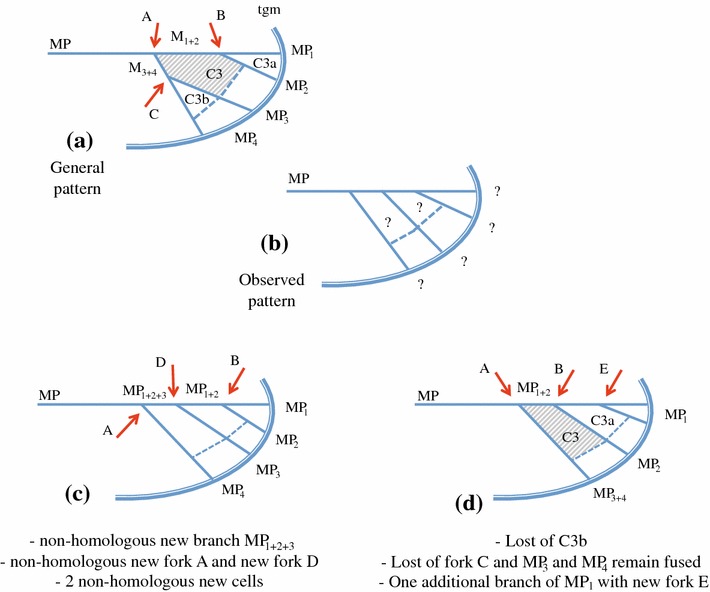
Interpretation of M branches and nodal cell C3 taking into account the branch terminals and topology (**c**) or the forking nodes (*red arrows*) (**d**) as landmarks (*tgm* tegmen margin). **a** General pattern and **b** observed pattern. **c**, **d** Alternative and conflicting interpretations of vein branches, forking nodes and cells; **d** is the interpretation retained
3. Separation of fused veins should be tested before suggesting new branch vein apparition. Even if additional terminal branches are not uncommon in planthopper tegmina, partial fusions are also commonly observed. Advocating a re-separation after fusion is more parsimonious than the loss of one branch and the appearance of a new one as illustrated in Fig. 7e–g. A relatively common case of vein fusion and re-separation in planthoppers is between M_3+4_ and CuA_1_ more or less at the nodal level such as in some Meenoplidae (Bourgoin 1997) or in some Lophopidae such as in *Magia*, Distant 1907 (Fig. 5d).

The Derbidae is probably the most interesting example of these difficulties as the taxa both addresses terminology and interpretation issues. For the former for instance, Zelazny (1981: Fig. 1) and Zelazny and Webb (2011: Figs. 122, 123) used the old terminology system (Ms, median sector) proposed by Muir (1917: Figs. 1–6) in Derbidae Rhotanini for the median branches (Ms1 = MP_3+4_; Ms1a, Ms1b = M_3_, M_4_; Ms2 = M_2_ and M = M_1_, according the system proposed in this paper). This leads to a misunderstanding of the homological corresponding structures out of this particular system and renders it difficult to directly share their data for enlarged taxonomical group studies, even just within the derbids themselves.

A more challenging issue in this group, however, remains the interpretation of the CuA–MP branches in several derbid tribes. Emeljanov (1994) reported the old controversy of opinions in the venation pattern between Muir (1918)—followed by Broomfield (1985) and Anufriev and Emeljanov (1988)—on one side and Synave (1973)—following Metcalf (1913) and followed by Dworakowska (1988) and Emeljanov (1994)—on the other side. In the first case, CuA is considered to fork several times before reaching the margin with more than two branches (Fig. 8a) sometimes with up to more than six branches according the taxa. In the second case, CuA forks only once into CuA_1_ and CuA_2_. The two branches extend to the margin according to Dworakowska (1988), and CuA_2_ fuses with PCu + A1 (Fig. 8b) as suggested by Synave (1973) or CuA_1_ and CuA_2_ fuse in a common stem reaching the margin (Fig. 8c) as according to Emeljanov (1994). Our recognition strategy agrees with this last interpretation and is illustrated in Fig. 8d. In this particular case, the areola postica is said to be closed. In a few other taxa, CuA_1_ and CuA_2_ might separate again and join the margin independently such as in *Achilixius* Muir 1923 (Achilixiidae) or *Caledonisia* Bourgoin 1997 (Meenoplidae).

**Fig. 7:**
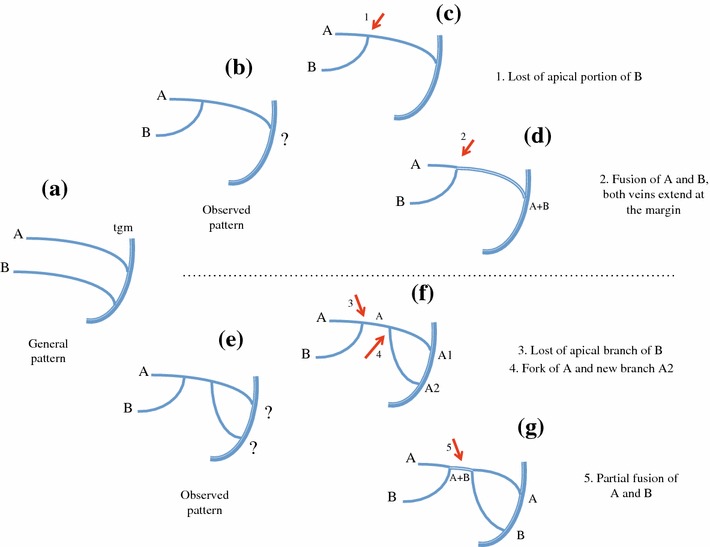
Schematic representation in the interpretation of modified patterns (**b)**, (**e**) derived from a generalized one (**a**). A partial (**g**) or full (**d**) coalescence of vein A and vein B is a more parsimonious explanation than the lost of the distal portion of vein B in (**c)**, (**f)** plus the emergence of a new branch A2 in (**f)**

Brachypterism and hyperpterism are here defined relatively to a Fulgoromorpha ground plan as established by Shcherbakov (1996). It is, however, important to observe that this ground plan is relatively simple and based on the a priori statement that Paraneoptera ancestors belong to the Palaeozoic Hypoperlida and that Hemiptera ancestors belong to Archescytinoidea (op. cit.), both groups having a simple venation pattern. But this ground pattern is in fact quite derived compared to the rich and complex venation observed in other Permian polyneopteran insect orders. It cannot be excluded that the first Hemiptera might have been present with a more complex/developed, but plesiomorphic, venation pattern such as in Aviorrhynchidae (Nel et al. 2013). Even if this happens, the three states of brachypterism, macropterism and hyperpterism could be maintained as a practical terminology system to define and describe precisely the tegmina evolution in planthoppers.

**Fig. 8:**
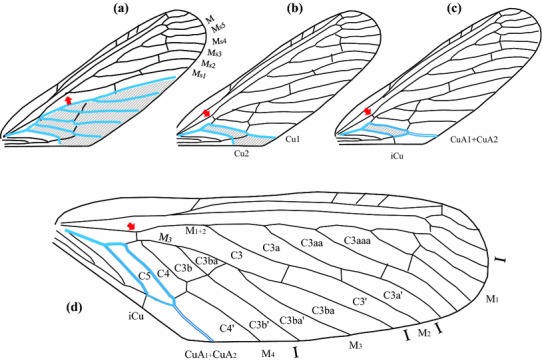
Conflicting interpretations of the cubital area (*shaded*) in the same Derbidae species: *Zoraida (Neozoraida) ugandensis* Distant, 1914. **a** According to Muir (1918) with a multibranched CuA (six branches in this example). **b**. According to Synave (1913) with two branches, Cu1 and Cu2, the latter fusing with the Y-vein (Cl1 + Cl2 in Synave terminology). The area postica is said ‘open’. **c** According to Emeljanov (1994) with two branches, CuA1 and CuA2, fusing into a common stem and extending to the margin to form the procubital cell. **d** Interpretation adopted in this paper, following Emeljanov’s interpretation, with M branches and nodal cells terminology. The area postica is said ‘closed’. *Red arrows* indicated the landmark of first M furcation

Tegmina reduction (brachypterism, micropterism) with all its transitions is usually paralleled by reduction of the tegulae, which in extreme cases, such as in strongly troglomorphic taxa (e.g. in Cixiidae, Meenoplidae, Kinnaridae and Delphacidae), can be entirely lost (Hoch 2002; Hoch et al. 2003, 2006). Brachypterism and micropterism are indeed most often observed in species which have adopted a cryptic way of life, such as in leaf litter, inside the soil or in caves (Hoch and Asche 1993; Hoch 1994, 2002; Hoch and Ferreira 2012, 2013). As tegmina (and wings) cease to be functional, venation pattern tends to show an increased intraspecific variation (Hoch 2002).

It is remarkable, though, that—while the hindwing can be entirely missing—there is not a single case known in the entire Fulgoromorpha where the tegmen has been completely reduced. It is conceivable that the presence of the tegmen (even if minute) is maintained by either an evolutionary constraint or selection: it may serve some sensory, acoustic or glandular function, or serve to cover spiracles to prevent excessive water loss in drier environments or may play a role in reproductive behaviour, all being different and non-exclusive reasons preventing a full apterism condition.

## Conclusions

For an analysis and understanding of the venation patterns, not only veins and veinlets, but also areas delimited by them should be taken into consideration. These latter are complex morphological characters (group of several more basic characters) that are generally most useful for identification purposes. However, they might be also used in phylogenies if cautiously analysed (in terms of homology), while indeed some of them are notable non-homologous morpho-functional structures (Wootton 1996) such as flexion lines and cross-veins alignments.

Working towards a standardized terminology of tegmina areas and veins (and more generally for any morphological structure) is important. It will strengthen the necessary quality of taxonomic descriptions. It is also obviously crucial when one wishes to establish morphological matrices based on homologous checked venation characters for phylogenetical reconstructions. Particularly for planthoppers, it will better address evolutionary scenarios of wing and tegmina transformations that took place over such a long period since the Carboniferous, which, because of fossils remains, are based on the only common dataset to share.

Finally, beside the single scientific issue, in the new era of on-line and open access data where published papers would become automatically e-checked and linked to new on-line identification keys and even used to e-fill directly big datasets with such kind of characters, sharing precisely defined homologous data through a standardized terminology (such as for these wing characters so widely used in the literature) will be a prerequisite. In that sense, this work is a first step towards an ontology allowing to formalize, to structure and to organize the information carried by the wings in Hemiptera through a controlled vocabulary.

## Footnotes

1 The latter term, used as 'coeliopterous', was subsequently used by Fennah (1982) to describe the tegmina of some tropiduchid tribes.
